# Modelling the asthma phenotype: impact of cigarette smoke exposure

**DOI:** 10.1186/s12931-018-0799-7

**Published:** 2018-05-10

**Authors:** Maria G. Belvisi, Katie Baker, Nicole Malloy, Kristof Raemdonck, Bilel Dekkak, Michael Pieper, Anthony T. Nials, Mark A. Birrell

**Affiliations:** 1 Respiratory Pharmacology, National Heart and Lung Institute, Faculty of Medicine, Imperial College London, Exhibition Road, London, SW7 2AZ UK; 2 Respiratory, Inflammation Autoimmunity RIA IMED Biotech Unit, AstraZeneca, Gothenburg, Sweden; 3 MRC and Asthma UK Centre in Allergic Mechanisms of Asthma, Imperial College London, London, UK; 4 Department of Anatomy, Faculty of Medicine, University of Porto, Alameda Prof. Hernâni Monteiro, 4200-319 Porto, Portugal; 5 Center for Health Technology and Services Research (CINTESIS), Faculty of Medicine, University of Porto, Rua Dr. Plácido da Costa, 4200-450 Porto, Portugal; 6 Boehringer Ingelheim Pharma GmbH & Co. KG, Rhein, Germany; 7 GSK, Stevenage, UK

**Keywords:** Asthma, Symptoms, Air pollution, Treatment, Cigarette smoke

## Abstract

**Background:**

Asthmatics that are exposed to inhaled pollutants such as cigarette smoke (CS) have increased symptom severity. Approximately 25% of adult asthmatics are thought to be active smokers and many sufferers, especially in the third world, are exposed to high levels of inhaled pollutants. The mechanism by which CS or other airborne pollutants alter the disease phenotype and the effectiveness of treatment in asthma is not known. The aim of this study was to determine the impact of CS exposure on the phenotype and treatment sensitivity of rodent models of allergic asthma.

**Methods:**

Models of allergic asthma were configured that mimicked aspects of the asthma phenotype and the effect of CS exposure investigated. In some experiments, treatment with gold standard asthma therapies was investigated and end-points such as airway cellular burden, late asthmatic response (LAR) and airway hyper-Reactivity (AHR) assessed.

**Results:**

CS co-exposure caused an increase in the LAR but interestingly attenuated the AHR. The effectiveness of LABA, LAMA and glucocorticoid treatment on LAR appeared to be retained in the CS-exposed model system. The eosinophilia or lymphocyte burden was not altered by CS co-exposure, nor did CS appear to alter the effectiveness of glucocorticoid treatment. Steroids, however failed to reduce the neutrophilic inflammation in sensitized mice exposed to CS.

**Conclusions:**

These model data have certain parallels with clinical findings in asthmatics, where CS exposure did not impact the anti-inflammatory efficacy of steroids but attenuated AHR and enhanced symptoms such as the bronchospasm associated with the LAR. These model systems may be utilised to investigate *how* CS and other airborne pollutants impact the asthma phenotype; providing the opportunity to identify novel targets.

## Background

Asthma is a respiratory disease that is increasing in prevalence globally. Airborne pollutants such as cigarette smoke (CS, direct and passive) and traffic/industrial pollution are reported to increase asthma susceptibility, cause quality of life issues and enhance symptom severity, frequency of attacks and disease exacerbations [1–23]. Smoking and passive smoking has also been shown to adversely impact on the effectiveness of standard treatment such as inhaled corticosteroid (ICS) in asthmatics [24–28] and worsen disease outcome [29]. Despite the fact that asthma is a severe and debilitating illness, a significant proportion of asthma patients smoke or are exposed to passive smoke [30]. As many as half of all adult asthma patients may be active, or previous smokers [13, 14]. Thus with the increase in airborne pollution levels, especially in developing countries, and continued exposure to CS (either directly or passively), it is important to try and understand the mechanism by which these pollutants impact on asthma pathogenesis and whether this contributes to treatment-resistance.

Within allergic asthma, exposure to allergen results in a biphasic bronchoconstrictor response. Immediate bronchoconstriction as a result of exposure is termed the Early Asthmatic Response (EAR) and typically occurs within 1 h of contact with aeroallergen. The Late Asthmatic Response (LAR) refers to a more prolonged bronchoconstriction event taking place approximately 3–8 h following contact with allergen. The LAR is often used within clinical studies exploring new therapeutic options with which to treat asthma and as such is considered to be a clinically relevant endpoint [11, 31].

Airway Hyper-Responsivity (AHR) is a cardinal feature of the asthma phenotype. It is defined as an increased sensitivity to inhaled stimuli resulting in narrowing of the airways, which would not usually occur in healthy individuals. This response manifests as excessive bronchoconstriction and airflow limitation, resulting in shortness of breath and chest tightness. Stimuli of AHR include pollution, allergens, cold air and spasmogens such as Methacholine (MCh). The endpoint of AHR in allergic asthmatics exposed to CS has been investigated but results are sparse and conflicting.

Many features of allergic asthma have been successfully modeled in rats and mice. The Brown Norway rat is considered to be one of the most suitable rat strain for use as an allergic asthma model. This particular strain is a high IgE producer, it produces robust responses to allergens (distinct EAR and LAR) and the infiltration of allergic airway inflammation is considered to be similar to that seen in asthmatic patients [31, 32]. The mouse is also considered to be an advantageous model of allergic asthma due to the possibility of the application of genetically modified (GM) strains and the fact that it comprises a highly characterised immune system.

The aim of this study was to determine the effect of CS co-exposure on the phenotype and treatment sensitivity of rodent models of allergic asthma. In order to investigate this, rodent models of allergic asthma were co-exposed to CS and endpoints of the Late Asthmatic Response (LAR), Airway Hyper-Responsivity (AHR) and airway cellular burden were assessed. The effectiveness of gold standard asthma treatments (i.e. ICS, LABA and LAMA) were also investigated within these models. It was hypothesised that the allergic asthma models exposed to CS would exhibit enhanced LAR and AHR responses and the efficacy of standard asthma treatments would be diminished within these groups.

## Methods

### Animals

All experimental protocols were approved by a local ethical review process and strictly adhered to the Animals (Scientific Procedures) Act 1986 UK Home Office guidelines and performed according to the ARRIVE guidelines. Male Brown Norway rats (200-250 g) and male C57BL/6 mice were obtained from Harlan, UK. All animals were housed in individually ventilated cages (IVC) and a 12-h light-dark cycle maintained. Prior to and during experimental periods, food and water was supplied ad libitum.

### Cigarette smoke exposure system

CS exposure was performed according to methods as previously described by our laboratory [33, 34]. Briefly, filtered research cigarettes (University of Kentucky Research Cigarettes, [Ref: #3R4F]) were stored at 4 °C, and 48 h prior to use they were brought to room temperature and the filters removed. On the day of exposures, the exposure system equipment was set up as previously described [33, 34] and the system flow set to 1.5 L/min. Animals were placed in stainless steel cages (rats: 12 per cage; mice: 40 per cage) before being placed into the system. Cigarettes were administered to the system via the pinch valve. Smoke exposure sessions lasted for 50 mins, defined as the last cigarette being removed from the system 50 mins from the first being lit. The system flow was checked at 15 min intervals, and the wellbeing of the animals checked continually through each session with the use of a torch for visibility. Total Suspended Particulate (TSP) was sampled at 30 min intervals in each exposure session. A filter membrane was weighed prior to being administered to the dry gas meter sampling unit. Each TSP sample period was 1 min. The filter was weighed again at the end of the sampling period. The dry gas meter recordings were noted at the start and end of each exposure period. TSP was calculated as follows:

$$ \mathsf{Particulate}\ \mathsf{weight}\ \left(\mathsf{mg}\right)=\mathsf{post}\ \mathsf{sampling}\ \mathsf{filter}\ \mathsf{weight}\hbox{--} \mathsf{pre}-\mathsf{sampling}\ \mathsf{filter}\ \mathsf{weight} $$

$$ \mathsf{Total}\ \mathsf{sample}\ \mathsf{volume}\ \left({\mathsf{m}}^3\right)=\mathsf{End}\ \mathsf{dry}\ \mathsf{gas}\ \mathsf{meter}\ \mathsf{reading}\hbox{--} \mathsf{start}\ \mathsf{dry}\ \mathsf{gas}\ \mathsf{meter}\ \mathsf{reading} $$

$$ \mathsf{TSP}\ \left(\mathsf{mg}/{\mathsf{m}}^3\right)=\mathsf{Particulate}\ \mathsf{Weight}\ \left(\mathsf{mg}\right)/\mathsf{Total}\ \mathsf{Sample}\ \mathsf{Volume}\ \left({\mathsf{m}}^3\right) $$

The TSP values were consistent and as such a consistent CS burden could be confirmed.

### Investigating the effect of cigarette smoke exposure in the Brown Norway rat model of the LAR

A rat model of allergic asthma was used as previously described [32]. Briefly, male Brown Norway rats were sensitised on day 0, 14 and 21 with chicken ovalbumin (OVA) (100 μg/rat, i.p., Grade V, Sigma, UK.) administered with Alum (20 mg/rat aluminium hydroxide and 20 mg/rat magnesium hydroxide, i.p., Alum™ Thermo Scientific, UK). Rats were exposed to room air or CS for 1 h, twice a day (4 h apart) on day 21, 22, 23, 24, 25, 26, and 27. On day 28 the rats were exposed to air/smoke in the morning and in the afternoon the rats were challenged with vehicle (saline, aerosolised for 30 min) or OVA (1% *w*/*v*, aerosolised for 30 min). The LAR was monitored in conscious BN rats for 1 to 6 h after challenge as previously described [31, 35]. The following day the animals were euthanised with pentobarbitone (200 mg/kg, i.p., Centaur Services, UK). Bronchoalveolar lavage (BAL) was carried out by injecting 3 ml of RPMI culture medium (Invitrogen, UK) via a cannula inserted into the trachea, waiting 30 s and then removing it. This was repeated and the collected BAL fluid (BALF) pooled. Total and type of white cells in the BALF were determined as previously described [32, 35].

### Assessing the effectiveness of standard asthma therapies in the CS co-exposed rat model of the LAR

To determine if CS co-exposure alters the effectiveness of current asthma therapies, rats were treated with topical glucocorticoid, budesonide; LABA, Olodaterol, and LAMA, glycopyrrolate. Briefly, under inhaled anaesthetic, rats (*n* = 8) received vehicle (0.5% ethanol in saline, 1 ml/kg, intratracheal), Olodaterol (1 mg/kg, dose selected from preliminary studies), budesonide (3 mg/kg, dose selected from previous work [35]) or glycopyrrolate (1 mg/kg, dose selected from preliminary studies) one hour before and 30 min after OVA challenge. LAR was measured in all three studies as described in the previous section and airway inflammation was assessed in the study with glucocorticoid intervention.

### Investigating the effect of cigarette smoke exposure in a C57BL/6 model of AHR

A mouse model that we have previously shown to feature AHR was applied to this body of work [36, 37]. Briefly, male C57bl/6 mice were sensitised on day 0 and 14 with either OVA (10 μg/ mouse, i.p.) with Alum™ (diluted 1:1 with saline, 100 μl i.p.) or HDM (0.5 μg/kg in 100 ul, i.p. from Greer, USA – No Alum™). On days 24, 25, 26 mice were challenged intranasally with vehicle (50 ul saline), OVA (2.5 mg/kg) or HDM (1.25 μg/kg). Purified HDM extract from Dermatophagoides pteronyssinus (Der p; lot number 124632; GREER laboratories, USA) with a known content of Der p1 (12.76 μg/mg dry weight) was used in these experiments. Endotoxin content – 125 EU/vial (121 μg HDM /vial).

Mice were exposed to room air or CS for 1 h, twice a day (4 h apart) on days 21 to 28. This CS exposure protocol was based on previous development work [33] using a system previously described [34]. On day 29 lung function (Penh) was assessed to increasing doses of inhaled spasmogen (aerosolised 5-HT) using a method previously described [36]. After the lung function had returned to pre-spasmogen levels the mice were euthanised with pentobarbitone (200 mg/kg, i.p., Centaur Services, UK). BAL was carried out by injecting 0.3 ml of RPMI culture medium (Invitrogen, UK) via a cannula inserted into the trachea, waiting 30 s and then removing it. This was repeated twice more and the collected BALF pooled. Total and type of white cells in the BALF was determined as previously described [36].

Assessing the effectiveness of steroid asthma therapy in the CS co-exposed mouse model of AHR.

To determine if CS co-exposure alters the effectiveness of glucocorticoids, mice received vehicle (0.5% methylcellulose plus 0.2% tween80 in water, 10 ml/kg, orally) or budesonide (0.3, 1 or 3 mg/kg, orally, doses selected from previous work [36]) twice per day on days 24–28 (1 h prior to the morning CS exposure and 1 h after the afternoon CS exposure). Airway cellular burden was assessed on day 29 as described above. AHR was not assessed because CS co-exposure attenuated the signal.

### Data analysis

Data are expressed as mean ± S.E.M. of n observations. Statistical significance was determined using either single or multiple comparisons (specific tests used are described in the Figure legends), using GraphPad Prism 5 software. A *P* value < 0.05 was taken as significant and all treatments were compared with the appropriate control group.

## Results

### Effect of cigarette smoke exposure on a rat model of allergic asthma

**Fig. 1:**
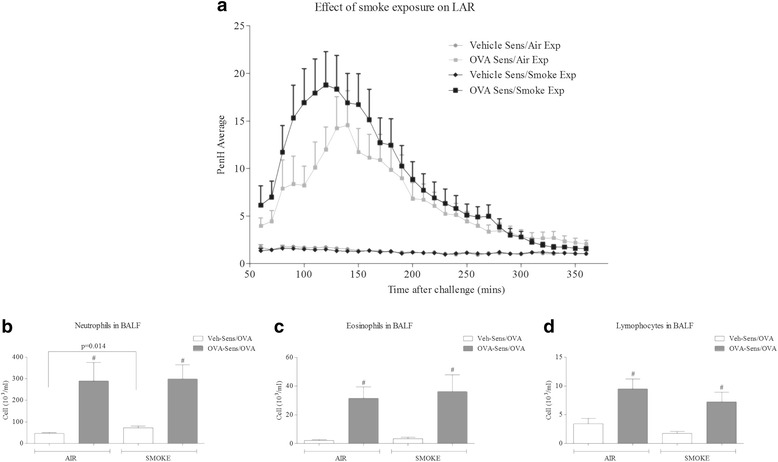
Effect of CS co-exposure on a rat model of allergic asthma. Sensitised male Brown Norway rats were challenged with an aerosol of saline or OVA for 30 min. Rats were co-exposed to room air or CS (twice a day) for eight days. Changes in lung function (Penh) were assessed from 1 h after the end of challenge for 5 h. BALF was collected the following day and the numbers of white cells assessed. Data are represented as mean ± S.E.M. for *n* = 8 animals in each group. **a**: LAR, **b**: neutrophil number, **c**: Eosinophil number and **d**: lymphocyte number. The statistical significance of the response to antigen and/or CS was determined using a Mann-Whitney U test and denoted with # (*P* < 0.05)

Antigen challenge led to a marked increase in respiratory distress (increased audible and visual signs) that correlated with a change in Penh levels in sensitised rats, as previously shown and described as a LAR [31, 35] (Fig. 1a). Exposure to CS alone appeared to have no effect compared to the appropriate control group, but when rats were co-exposed with the antigen there was an increase in the magnitude of the LAR (Fig. 1a).

The day after antigen challenge, we observed a significant increase in neutrophils, eosinophils and lymphocytes in the BALF (Fig. 1b-d). CS alone caused a small but statistically significant increase in BALF neutrophilia (Fig. 1b), but did not alter the allergic cellular inflammation triggered with antigen challenge (Fig. 1b-d).

**Fig. 2:**
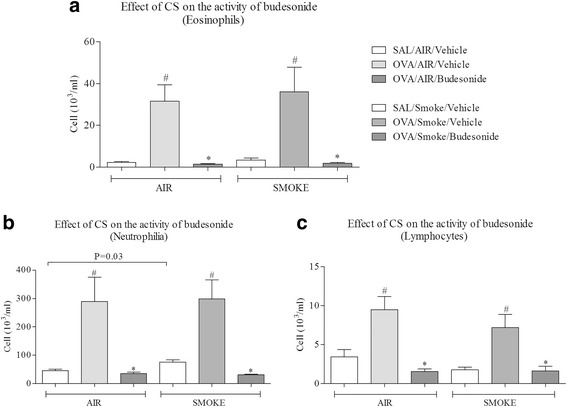
Effect of glucocorticoid treatment on a CS co-exposure rat model of allergic asthma. Sensitised male Brown Norway rats were challenged with an aerosol of saline or OVA for 30 min. Rats were co-exposed to room air or CS for eight days and dosed with vehicle (1 ml/kg, intratracheal) or budesonide (3 mg/kg, i.t) one hour prior to and 30 min after antigen challenge. BALF was collected the following day and the numbers of white cells assessed. Data are represented as mean ± S.E.M. for *n* = 8 animals in each group. **a**: Eosinophil number, **b**: neutrophil number and **c**: lymphocyte number. The statistical significance of the response to antigen and/or CS was determined using Mann-Whitney and denoted with # (*P* < 0.05). The significance of the impact of budesonide was determined using Mann-Whitney and denoted with * (*P* < 0.05)

To determine if the CS plus antigen challenge phenotype had an altered sensitivity to gold standard asthma treatment we profiled a topical glucocorticoid, LABA and LAMA. Figure 2 shows that antigen challenge increased the BALF levels of eosinophils, neutrophils and lymphocytes. CS alone increased neutrophil number but did not significantly alter the response to antigen (Fig. 2). Topical treatment with the clinically relevant glucocorticoid, budesonide, inhibited the cellular inflammation in both the antigen alone and the antigen plus CS co-exposed animals (Fig. 2). This would suggest that co-exposure with CS did not alter the anti-inflammatory effectiveness of budesonide within this model.

**Fig. 3:**
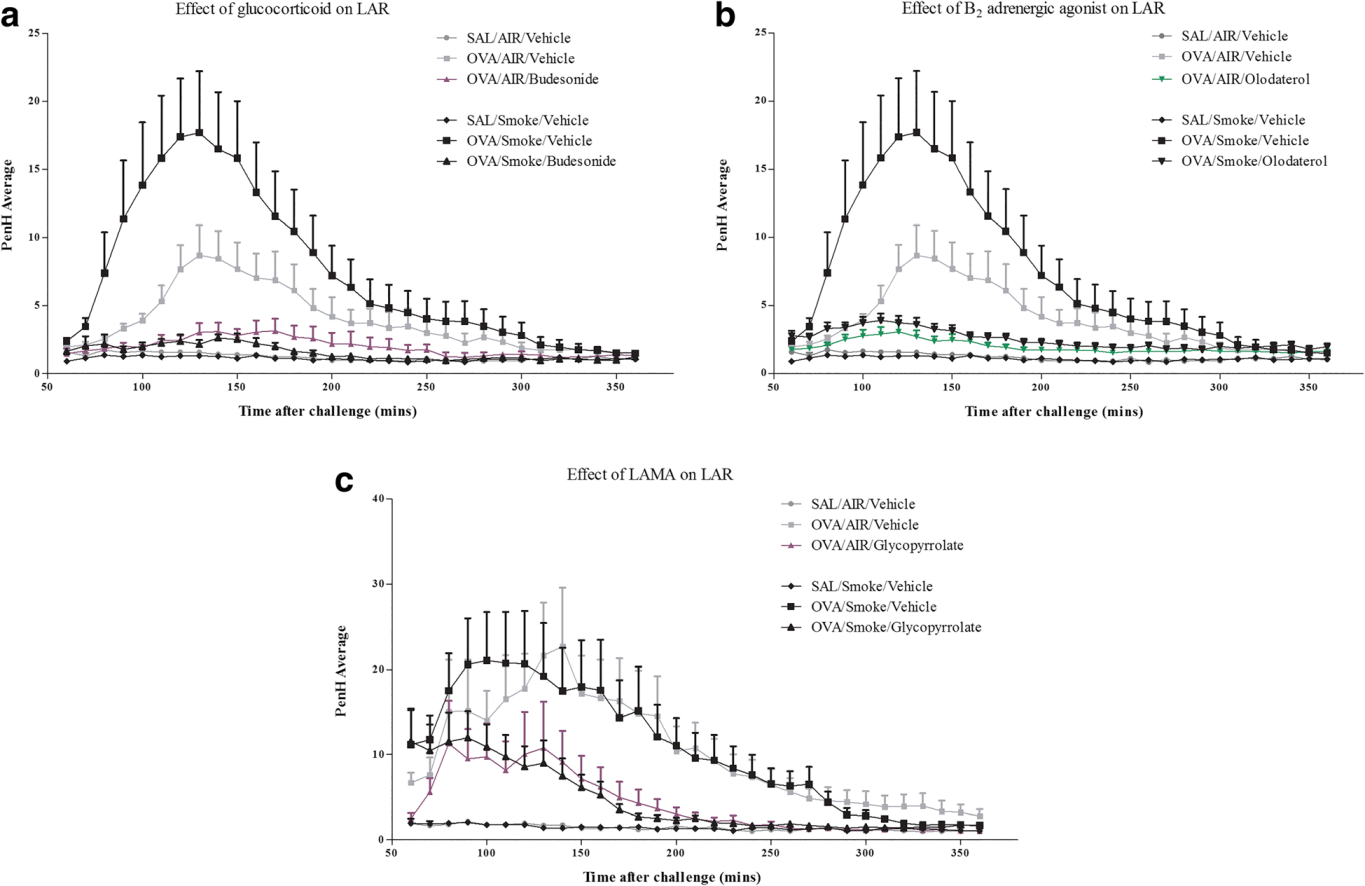
Effect of gold standard asthma treatments on a CS co-exposure rat model of allergic asthma. Sensitised male Brown Norway rats were challenged with an aerosol of saline or OVA for 30 min. Rats were co-exposed to room air or CS for eight days and dosed with vehicle (1 ml/kg, intratracheal, i.t.), budesonide (3 mg/kg, i.t), Olodaterol (1 mg/kg, i.t.) or glycopyrrolate (1 mg/kg, i.t.) one hour prior to and 30 min after antigen challenge. Changes in lung function were assessed from 1 h after the end of challenge for 5 h. Data are represented as mean ± S.E.M. for *n* = 8 animals in each group. **a**: Glucocortoid, **b**: LABA and **c**: LAMA

Treatment with topical glucocorticoid, LABA and LAMA impacted on the LAR observed after antigen challenge (Fig. 3). CS exposure alone appeared to have no direct effect on changes in Penh but co-exposure increased the LAR. The CS co-exposed LAR signal was almost completely blocked by treatment with olodaterol or glucocorticoid and attenuated by glycopyrrolate (Fig. 3). This would indicate that although co-exposure with CS leads to an enhanced LAR in this model, this particular asthma phenotype is still sensitive to topical glucocorticoid and bronchodilator treatment.

### Effect of cigarette smoke exposure on mouse models of allergic asthma

**Fig. 4:**
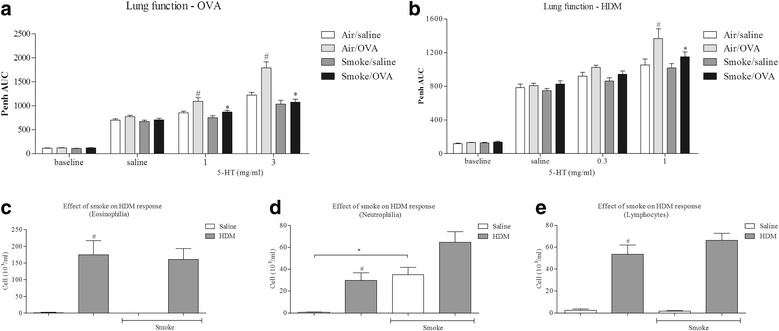
Effect of CS co-exposure on mouse models of allergic asthma. Sensitised male mice were challenged with intranasal saline (50 ul) or antigen (OVA or HDM) once a day for 3 days. Mice were co-exposed to room air or CS for eight days. Changes in airway reactivity (AR) to inhaled 5-HT were assessed 3 days after the final antigen challenge. BALF was then collected and the numbers of white cells assessed. Data are represented as mean ± S.E.M. for n = 8 animals in each group. **a**: AR after OVA challenge, **b**: AR after HDM **c**: Eosinophil number, **d**: Neutrophil number and **e**: lymphocyte number. The statistical significance of the response to antigen and/or CS was determined using a Mann-Whitney U test and denoted with # (*P* < 0.05)

Exposure to antigen, either OVA or HDM, resulted in AHR to the inhaled spasmogen (5-HT) (Fig. 4a and b). Intriguingly, whilst exposure to CS alone did not appear to alter responses to 5-HT, in both model systems CS co-exposure attenuated the AHR. Antigen challenge caused a significant increase in BAL eosinophils, neutrophils and lymphocytes (Fig. 4c, d and e). CS alone significantly increased neutrophil number in the BAL but did not alter the level of eosinophils and lymphocytes in BAL after HDM challenge. An additive effect was observed for the neutrophilic inflammation after combined HDM and CS challenge (Fig. 4).

**Fig. 5:**
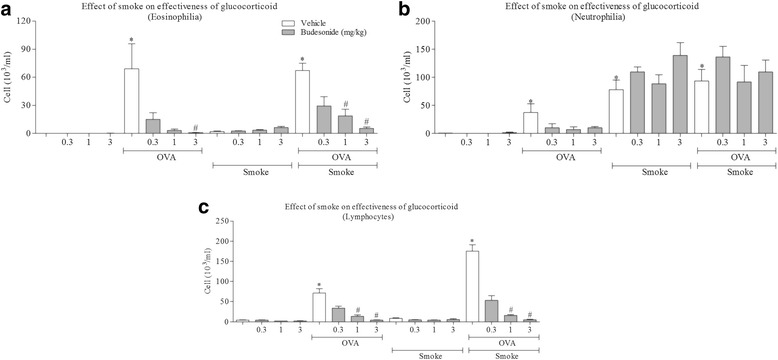
Effect of glucocorticoid treatment on CS co-exposure mouse models of allergic asthma. Sensitised male mice were challenged with intranasal saline (50 ul) or antigen (OVA or HDM) once a day for 3 days. Mice were co-exposed to room air or CS for eight days. BALF was then collected and the numbers of white cells assessed. Mice were dosed with vehicle (10 ml/kg, orally, p.o.) or budesonide (3 mg/kg, p.o.) one hour prior to the morning CS challenges and one hour after afternoon CS challenges. Data are represented as mean ± S.E.M. for n = 8 animals in each group. **a**: Eosinophil number, **b**: Neutrophil number and **c**: lymphocyte number. The statistical significance of the response to antigen and/or CS was determined using a Mann-Whitney U test and denoted with * (*P* < 0.05). The significance of the impact of budesonide was determined using one way ANOVA followed by a Bonferoni’s correction post-test # (*P* < 0.05)

As CS co-exposure attenuated AHR in both model systems, we could not determine the impact of standard asthma therapies on this end point. Therefore we profiled the anti-inflammatory effects of a glucocorticoid (budesonide, administered p.o.) on the cellular inflammation only. As can be seen in Fig. 5, antigen challenge increased BAL cellular inflammation, and this signal was inhibited by treatment with budesonide. Co-exposure to CS did not appear to impact on the effectiveness of budesonide treatment on antigen induced increase in eosinophil and lymphocyte numbers (Fig. 5). Neutrophil numbers after CS challenge alone, or in combination with antigen challenge, was not altered by budesonide treatment as previously reported [38].

## Discussion

Airborne pollutants such as CS (direct and passive) are known to increase asthma symptoms, severity, frequency of attacks and disease exacerbations and to adversely impact the effectiveness of standard treatment such as inhaled corticosteroid (ICS) in asthmatics. Despite this, the levels of smoking in asthmatic patients are still high; with some estimates suggesting that smoking asthmatics in developed countries represent approximately one quarter of all sufferers. Thus it is important to try and understand the mechanism by which pollution impacts on asthma pathogenesis and treatment. To investigate this effect we determined how CS altered the asthma phenotype in rodent models of allergic asthma. Our studies showed that CS co-exposure increased the magnitude of the LAR, but actually inhibited the AHR signal. CS co-exposure did not appear to impact on cellular burden (above and beyond an additive effect) or treatment effectiveness. This is the first pre-clinical study to comprehensively examine the impact of CS co-exposure on the asthmatic phenotype, and the data demonstrates that these models have many parallels with clinical observations suggesting their usefulness for future investigations.

Antigen challenge triggered cellular recruitment in sensitised animals as previously reported [35, 39]. Similarly exposure to CS caused the expected increase in airway neutrophilia [40]. Co-exposure of the allergic asthma models with CS appeared not to alter the cellular profile above and beyond an additive effect (i.e. neutrophil number). Similar increases in neutrophil numbers have been reported in asthmatics that smoke [41, 42] and it is believed that this cell type plays an important role in the pathophysiology of asthma and is linked to the “asthma COPD overlap syndrome”. Furthermore, Meghji et al. have recently shown similar eosinophilia data in human asthmatics demonstrating that smoking status does not alter the levels following antigen challenge [43]. Interestingly there are some reports that eosinophil numbers are reduced in asthmatics that smoke [14, 44]. This observation could depend on a number of factors including the level of smoke exposure/pack years, asthmatic status and time of sampling. The published preclinical data from studies examining the effect of CS co-exposure is varied, with some reporting reductions and others augmentation in cellular inflammation (the main focus is often eosinophil numbers) [45–59]. These disparate findings appear to be largely due to variations in CS co-exposure protocols.

Treatment with a clinically relevant corticosteroid, budesonide, inhibited the allergen induced cellular inflammation in the model systems as expected [35, 36], whilst it failed to impact on the CS induced neutrophilia as previously shown [38, 60, 61]. In our studies, co-exposure with CS did not appear to impact the effectiveness of budesonide treatment, a similar result was published by Song et al. [59]. Surprisingly few clinical studies have described the effects of steroid treatment on airway inflammation in smoking asthmatics; the studies tend to report lung function or asthma control as the primary endpoint. In addition, if pulmonary cellular inflammation is described, it is typically only eosinophilia that is reported, therefore there is little direct evidence on the effects of steroids on other inflammatory cells in smoking asthmatics. ICS have been shown to reduce sputum eosinophils in asthmatics, but not in smoking asthmatics in short term and long term studies [62], but others have shown that ICS do improve sputum eosinophils and ECP in smokers and non-smokers alike [29]. Therefore, the effect of smoking on the anti-inflammatory effects of steroids in asthmatics is currently controversial.

A striking observation is the apparent blockade of AHR in the model systems, whether it was driven by an allergic response to OVA or HDM. A similar finding was recently reported in asthmatic smokers that were exposed to a range of antigens and challenged with inhaled MCh [43]. As stated by the authors, it is not clear what the clinical significance is of this observation. One could speculate that as it is well known that smoking does increase clinical symptoms, the measurement of airway reactivity could be clinically irrelevant. Another group has reported that smoke challenge increases AHR in asthmatics but these experiments were performed using a sub-population of asthmatics that have previously reported to be sensitive to CS [22, 23]. Furthermore, the change was observed in only 30% of this sub-population and a similar number were affected in non-asthmatics. Other pre-clinical studies have reported similar findings with CS co-exposure inhibiting the AHR [54, 55]. Currently the mechanism by which CS causes this effect is not known. Melgert et al. (2004) suggested it was through the reduction of cellular inflammation in their model, but this seems unlikely as in our model systems since cellular inflammation was not decreased. There has been some speculation as to whether CS could be directly or indirectly evoking bronchodilation. Indeed CS is known to contain carbon monoxide which has been reported to reduce mouse AHR [63]; furthermore, CS can induce the release of bronchodilation substances such as PGE_2_ and nitric oxide [64]. In addition, CS contains nicotine, which conceivably could alter AHR. We believe, however, that these mechanisms are unlikely as normal airway reactivity to inhaled spasmogen was not altered by CS exposure, and the model systems presented with a strong LAR signal. Both these end points should be altered if CS was causing bronchodilation. Other possible mechanisms by which CS co-exposure reduces the AHR signal could be through the reduction of the mediators driving the AHR and the many cytokines suggested to be involved such as IL-5, IL-13 and IL-17 [65–71] or the production of mediators reported to inhibit AHR like TGFb [72, 73]. Indeed it has been reported that CS co-exposure increases levels of TGFb [74]. Unfortunately measurement of these end points is not possible in our studies as they were designed to focus on cellular inflammation and AHR, and not cytokine levels (the optimum time for cytokine measurements is much earlier) [75]. Another possible mechanism by which CS alters AHR could be due to an impact on airway smooth muscle (ASM), either the increased ability to contract [76] or the remodelling changes reported such as increased ASM thickness via antigen induced increase in proliferation/migration associated with the AHR phenotype [77]. Of the published studies, some have suggested CS increases proliferation, some have suggested inhibition and others to modulate the contractile response, thus this mechanism is still a possibility but needs to be further investigated [78–86]. Finally, CS could be causing remodelling in the airway which subsequently impacts on AHR. Indeed it has been reported that CS increases airway remodelling in pre-clinical asthma models [48, 52, 87].

Despite the loss of the AHR phenotype in the models following CS exposure, the LAR remains a clear feature; a similar observation was made in smoking human asthmatics [43]. Indeed, our data suggests that CS co-exposure actually enhances this cardinal feature of asthma. It is therefore tempting to speculate that it is this symptom of asthma that is central to the detrimental impact CS has on asthmatics.

As far as we know, we are the first to examine the effect of CS co-exposure on the LAR in a preclinical model. It is currently not clear how CS is exacerbating the LAR signal. One could speculate that as previous data has strongly implicated the TRPA1 - sensory nerve – parasympathetic axis in the LAR [31] that CS is somehow modulating elements of this pathway. Indeed it is well known that CS contains elements like acrolein which can activate TRPA1 [88, 89]. Further, TRPA1 is the molecular target for by-products of oxidative stress including Reactive Oxygen Species (ROS) and other electrophilic compounds, including hypochlorite and hydrogen peroxide which are linked to CS exposure [90–94]. As CS alone did not cause a “LAR” like response, it would seem that CS induced exacerbation of the response is not simply due to an increase of TRPA1 activator(s). One possible reason for the synergy between CS and antigen challenge could be that CS is increasing the sensitivity of airway sensory nerves to TRPA1 activators. Indeed we, and others, have observed that CS exposure can increase sensory nerve responses to TRPV1 ligands [95], furthermore we have unpublished data that suggests that TRPA1 responses are also increased. It is interesting to note that whilst we do not yet know the mechanism by which CS exacerbates LAR, current therapies such as ICS and LABA can combat this symptom of asthma. Furthermore, the inhibition of LAR in this model with glycopyrrolate confirms previous finding using another LAMA, tiotropium [31, 96].

## Conclusion

The aim our investigation was to determine the effect of CS co-exposure on the phenotype and treatment sensitivity of rodent models of allergic asthma. In order to investigate this, rodent models of allergic asthma were co-exposed to CS and endpoints of the Late Asthmatic Response (LAR), Airway Hyper-Responsivity (AHR) and airway cellular burden were assessed. The impact of ICS, LAMA and LABA were also observed within these models.

In summary, we found that the magnitude of LAR within the allergen sensitised models increased with co-exposure to CS and is concordant with our initial hypothesis. Divergent with our hypothesis; ICS, LAMA and LABA attenuated the LAR across both CS exposed and non-exposed groups. Interestingly the AHR was attenuated with exposure to CS. This was accompanied by an increase in neutrophilic inflammation, and although ICS was successful in attenuating overall cellular inflammation, the enhanced neutrophil populations observed remained undiminished.

We suggest that the data from these studies have parallels with clinical findings and that these model systems may be useful tools in helping to understand how exposure to airborne pollutants such as CS can alter the asthmatic phenotype. We propose that these model systems will be extremely useful in future research and will provide the opportunity to identify novel targets for asthma.

## Abbreviations

AHR: Airway hyperresponsiveness
Alum: 20 mg/ml aluminium hydroxide and 20 mg/ml magnesium hydroxide
AUC: Area under the curve
BALF: Bronchoalveolar lavage fluid
i.p.: Intraperitoneal
i.t.: Intratracheal
IgE: Immunoglobulin E
LAR: Late asthmatic response
OVA: Ovalbumin
PBS: Phosphate buffered saline
Penh: Enhanced pause
S.E.M.: Standard error of the mean
WBP: Whole body plethysmograph
